# Risk Assessment for Highly Pathogenic Avian Influenza A(H5N6/H5N8) Clade 2.3.4.4 Viruses

**DOI:** 10.3201/eid2710.210297

**Published:** 2021-10

**Authors:** Christine H.T. Bui, Denise I.T. Kuok, Hin Wo Yeung, Ka-Chun Ng, Daniel K.W. Chu, Richard J. Webby, John M. Nicholls, J.S. Malik Peiris, Kenrie P.Y. Hui, Michael C.W. Chan

**Affiliations:** The University of Hong Kong, Hong Kong, China (C.H.T. Bui, D.I.T. Kuok, H.W. Yeung, K.-C. Ng, D.K.W. Chu, J.M. Nicholls, J.S.M. Peiris, K.P.Y. Hui, M.C.W. Chan);; St. Jude Children’s Research Hospital, Memphis, Tennessee, USA (R.J. Webby)

**Keywords:** influenza, human airway organoids, risk assessment, tropism, innate host responses, HPAI H5Nx, clade 2.3.4.4, alveolar epithelial cells, viruses, Hong Kong, China

## Abstract

The numerous global outbreaks and continuous reassortments of highly pathogenic avian influenza (HPAI) A(H5N6/H5N8) clade 2.3.4.4 viruses in birds pose a major risk to the public health. We investigated the tropism and innate host responses of 5 recent HPAI A(H5N6/H5N8) avian isolates of clades 2.3.4.4b, e, and h in human airway organoids and primary human alveolar epithelial cells. The HPAI A(H5N6/H5N8) avian isolates replicated productively but with lower competence than the influenza A(H1N1)pdm09, HPAI A(H5N1), and HPAI A(H5N6) isolates from humans in both or either models. They showed differential cellular tropism in human airway organoids; some infected all 4 major epithelial cell types: ciliated cells, club cells, goblet cells, and basal cells. Our results suggest zoonotic potential but low transmissibility of the HPAI A(H5N6/H5N8) avian isolates among humans. These viruses induced low levels of proinflammatory cytokines/chemokines, which are unlikely to contribute to the pathogenesis of severe disease.

The genetic evolution of the highly pathogenic avian influenza (HPAI) subtype H5N1 A/goose/Guangdong/1/1996 lineage has resulted in the divergence and generation of 10 distinct virus clades (0–9) and multiple subclades (*1*,*2*). Since early 2014, novel reassortant HPAI A(H5N6/H5N8) viruses of clade 2.3.4.4 have gained attention because of their rapid evolution and global spread. They have been widely distributed among regions of Asia, Europe, and Africa and have been reported in North America (mainly in the United States and Canada), accompanied by further evolution into subclades 2.3.4.4a–h (*3*). Many avian species, including wild aquatic birds, domestic poultry, and zoo birds, are susceptible to the infection or support transmission of clade 2.3.4.4 viruses, resulting in unprecedented panzootic waves accompanied by massive culling and major economic losses to the poultry industry (*1*,*2*). Detection of HPAI H5N6 clade 2.3.4.4 viruses in cats, pigs, and humans has also been reported (*4**–**6*). As of May 2021, there have been 29 laboratory-confirmed cases of human infection, including at least 16 deaths (*3*,*6**–**8*). The isolated viruses mainly belong to subclades 2.3.4.4a, b, d, g, and h. These cases in humans were mainly sporadic and linked to direct contact with poultry or contaminated poultry market environments (*7*). The initial clinical signs in hospitalized patients were influenza-like, followed by severe pneumonia, acute respiratory distress syndrome, and multiple organ failure in deceased patients (*7*,*8*). Natural infection of mammals by HPAI H5N8 clade 2.3.4.4 viruses is not as commonly reported.

In February 2021, the Russian Federation reported detecting 7 cases of asymptomatic human infection with HPAI H5N8 clade 2.3.4.4b viruses in poultry farm workers, linked to a poultry outbreak (*9*). However, a serologic study revealed the presence of antibodies to an HPAI H5N8 clade 2.3.4.4 virus in 61 of 760 serum samples from persons who had had contact with infected or deceased birds during the 2016–17 HPAI outbreaks in Russia (*10*), providing evidence of overlooked human infection.

The persistent circulation of clade 2.3.4.4 viruses among bird populations enables continuous reassortment with prevailing low pathogenicity avian influenza (LPAI) viruses. Together with intercontinental dissemination through wild aquatic bird migration and potential interspecies transmission leading to mammalian adaptation, this circulation poses a major risk to human health should clade 2.3.4.4 viruses gain efficient human-to-human transmissibility, especially when immunity in the general population is lacking (*1*,*2*,*8*). Some HPAI H5N6/H5N8 clade 2.3.4.4 viruses have been shown to bind both α2,6- (human) and α2,3- (avian) linked sialic acid receptors, and more than half of HPAI H5N6 clade 2.3.4.4 isolates from humans found in GISAID acquired E627K or D701N substitutions in the polymerase basic (PB) 2 protein, which play prominent roles in mammalian adaptation of avian influenza viruses (*8*,*11**–**13*). An HPAI H5N8 clade 2.3.4.4 virus quickly acquired virulence markers, enhancing its virulence in mice and replication and polymerase activity in human cell lines within 5 murine passages (*14*), suggesting potential rapid adaptation after repeated virus introduction.

Surveillance efforts and characterization of clade 2.3.4.4 viruses provide insight into their pathogenicity and transmissibility, which can be used to prevent future outbreaks and assess zoonotic potential. Experimentally inoculated ferrets, mice, and guinea pigs displayed considerable variation in pathogenicity, and transmission by direct contact was demonstrated in guinea pig and ferret models (*8*,*11**–**13*,*15*,*16*). A few studies have also indicated efficient replication of HPAI H5N6 clade 2.3.4.4 viruses in human bronchus and lung explants and in primary human bronchial epithelial cells, which might have been linked to their respective successful infection of humans and direct-contact transmission among ferrets (*16*,*17*).

To further build on these findings, we used physiologically relevant 3-dimensional human airway organoids and primary human alveolar epithelial cells to investigate the tropism and innate host responses of 5 HPAI H5N6/H5N8 clades 2.3.4.4b, e, and h avian isolates from 2016–2018. We compared these responses to those of earlier human isolates of HPAI H5N1 clades 0 and 2.3.2.1b, HPAI H5N6 clade 2.3.4.4, an influenza A(H1N1)pdm09 virus (pH1N1), and an LPAI H5N8 virus.

## Methods

### Viruses

We used 3 HPAI H5N6 avian isolates: A/environment/Hong Kong/WCRB-01/2018 (avHPAI H5N6/DK01) of clade 2.3.4.4h, isolated from the outside of a chilled duck (GISAID accession no. EPI_ISL_885144); A/spoonbill/HK/17-18259/2017 (avHPAI H5N6/18259) of clade 2.3.4.4b, isolated from a trachea tissue sample of a dead black-faced spoonbill (GISAID accession no. EPI_ISL_885145); and A/northern pintail/HK/MP692/2016 (avHPAI H5N6/MP692) of clade 2.3.4.4e, isolated from a fecal sample of a northern pintail (GISAID accession no. EPI_ISL_885147). We also used 2 HPAI H5N8 clade 2.3.4.4b avian isolates: A/chicken/Egypt/F1366A/2017 (avHPAI H5N8/636099) (GISAID accession no. EPI_ISL_885148) and A/grey-headed gull/Uganda/200144/2017 (avHPAI H5N8/642613) (GISAID accession no. EPI_ISL_885149); 1 HPAI H5N6 clade 2.3.4.4 human isolate A/Guangzhou/39715/2014 (HPAI H5N6/39715) from the throat swab of a 59-year-old male patient on day 8 of illness (GenBank accession no. KP765785-KP765792); 2 HPAI H5N1 human isolates, A/Hong Kong/483/1997 (HPAI H5N1/483) of clade 0 isolated from a person with a fatal case (GenBank accession nos. GU052096-GU052104, AF258820, AF084277) and A/Shenzhen/1/2011 (HPAI H5N1/SZ1) of clade 2.3.2.1b (GISAID accession no. EPI_ISL_891209); LPAI H5N8 avian isolate A/northern pintail/Hong Kong/MP5883/2004 (avLPAI H5N8/MP5883) (GISAID accession no. EPI_ISL_885151); and pH1N1 virus A/Hong Kong/415742/2009. 

We prepared virus stocks in MDCK cells with limited passages. To determine virus titers, we used 50% tissue culture infectious dose (TCID_50_) assays.

### Human Airway Organoids

We cultured human airway organoids from cells isolated from human lung tissue and infected in 6 log TCID_50_/mL virus for 1 h at 37°C as previously described (*18*,*19*). We collected supernatant at 1, 24, 48, and 72 h after infection for virus titration by TCID_50_ assay. We fixed organoids in 4% paraformaldehyde at postinfection hours 24 and 48 for immunohistochemical double staining and collected cell lysates at postinfection hour 24 for measurement of mRNA expression.

### Primary Human Alveolar Epithelial Cells

We isolated alveolar epithelial cells from human lung tissues, cultured, and infected at multiplicities of infection (MOIs) of 0.01 and 2 for 1 h at 37°C as previously described (*17*,*18*). We collected supernatant at 1, 24, 48, and 72 h after infection for virus titration by TCID_50_ assay and collected cell lysates at 24 h after infection to measure mRNA expression. We compiled a description of our detailed study methods (Appendix 1).

## Results

### Productive Replication 

All 5 HPAI (H5N6/H5N8) avian isolates demonstrated productive replication in human airway organoids and alveolar epithelial cells (MOI 0.01); by 72 h after infection, mean peak titers were 3.7–5.1 log TCID_50_/mL for human airway organoids and 4.6–7.0 log TCID_50_/mL for alveolar epithelial cells (Figure, panels A, B). Mean peak titers of HPAI H5 isolates from humans were 5.0–6.7 log TCID_50_/mL for human airway organoids and 6.5–7.7 log TCID_50_/mL for alveolar epithelial cells. As expected, pH1N1 replicated to the highest mean peak titer of 7.1 log TCID_50_/mL in human airway organoids but reached only 2.5 log TCID_50_/mL in alveolar epithelial cells. AvLPAI H5N8/MP5883 replicated to a mean peak titer of 3.5 log TCID_50_/mL in human airway organoids but did not show any detectable replication in alveolar epithelial cells. When we compared the replication kinetic areas under the curve (AUCs), which estimated the total quantity of virus released, we found comparable AUC values among the HPAI H5N6/H5N8 avian isolates but lower AUC values for HPAI H5N6/H5N8 avian isolates than for the HPAI H5N6/39715 human isolate in human airway organoids and in alveolar epithelial cells (Figure, panels C, D). The AUC values of HPAI H5N6/H5N8 avian isolates were also lower than those of pH1N1 in human airway organoids and the HPAI H5N1/483 human isolate in alveolar epithelial cells, except for the statistically insignificant AUC values between avHPAI H5N6/18259 and HPAI H5N1/483 in alveolar epithelial cells.

**Figure:**
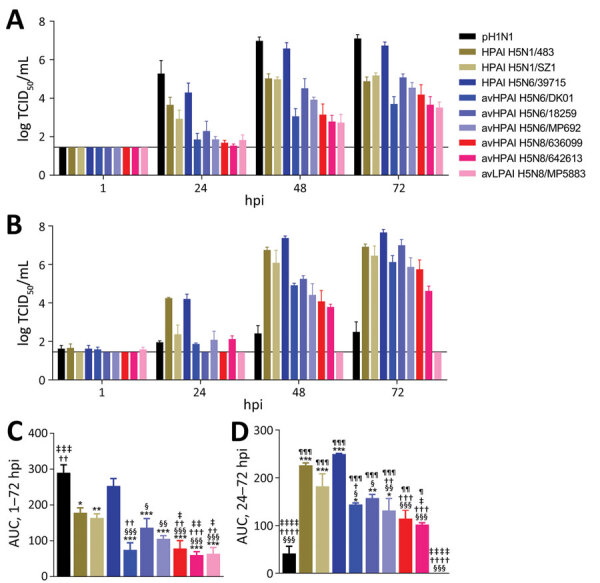
Replication kinetics of influenza A viruses. A, B) Replication in human airway organoids infected with 6 log TCID_50_/mL virus (A) and primary human alveolar epithelial cells infected at multiplicity of infection 0.01 at 37°C (B). Virus titers in culture medium (mean ± SEM, n>3) were determined by TCID_50_ assays with a detection limit of 1.5 log TCID_50_/mL, denoted by a solid line. Statistical significance between virus titers at each time point after infection is provided in Appendix 1 Figure. C, D) The areas under the replication kinetic curves above the detection limit in human airway organoids from 1 to 72 hpi (C) and alveolar epithelial cells from 24 to 72 hpi (mean ± SEM, n >3) (D). Statistical significance between AUC values was analyzed by using 1-way analysis of variance with Bonferroni posttests. *p<0.01; **p<0.001; ***p<0.0001 (compared with pH1N1); †p<0.05; ††p<0.01; †††p<0.001; ††††p< 0.0001 (compared with HPAI H5N1/483); ‡p<0.05; ‡‡p<0.01; ‡‡‡p< 0.001; ‡‡‡‡p<0.0001 (compared with HPAI H5N1/SZ1); §p<0.01; §§p<0.001; §§§ p<0.0001 (compared with HPAI H5N6/39715); ¶p<0.01; ¶¶p< 0.001; ¶¶¶p<0.0001 (compared with avLPAI H5N8/MP5883). AUC, area under the curve; av, avian; HPAI, highly pathogenic avian influenza; hpi, hours postinfection; LPAI, low pathogenicity avian influenza; pH1N1, influenza A(H1N1)pdm09 virus; TCID_50_, 50% tissue culture infectious dose.

### Cellular Tropism 

According to immunohistochemistry double staining, avHPAI H5N6/18259, avHPAI H5N6/MP692, and avHPAI H5N8/636099 infected acetyl-α-tubulin–positive ciliated cells, SCGB1A1–positive/CC10–positive secretory club cells, MUC5AC–positive secretory goblet cells, and p63-α–positive basal cells, similar to pH1N1, HPAI H5N1/483, HPAI H5N6/39715, and avLPAI H5N8/MP5883 (Appendix 2 Figure 1). avHPAI H5N6/DK01 and HPAI H5N1/SZ1 infected ciliated cells, club cells, and goblet cells. avHPAI H5N8/642613 infected only club cells.

### Proinflammatory Cytokine and Chemokine Induction

We studied induction of proinflammatory cytokines and chemokines by using HPAI H5N1/483 as a high inducing virus control and pH1N1 as a low inducing virus control. At 24 h after infection, HPAI H5N1/483 tended to induce higher mRNA levels of IFN-β, IFN-λ1, CCL5, CXCL10, TNFα, IL-6, ISG15, and MX1 than most HPAI H5N6/H5N8 avian isolates in human airway organoids and in alveolar epithelial cells (MOI 2); we observed statistical significance for IFN-λ1, CXCL10, and MX1 in human airway organoids and IFN-β, IFN-λ1, CCL5, CXCL10, ISG15, and MX1 in alveolar epithelial cells (Appendix 2 Figure 2). We found no statistically significant differences in the mRNA levels of these genes between HPAI H5N6/H5N8 avian isolates, HPAI H5N6/39715, pH1N1, and avLPAI H5N8/MP5883 in human airway organoids. Similarly, we detected only a few differences between their IFN-β, TNFα, ISG15, and MX1 mRNA levels in alveolar epithelial cells.

### Molecular Comparisons 

Molecular analysis revealed that all 5 HPAI H5N6/H5N8 avian isolates and the 3 HPAI H5 human isolates contained a 5 to 6 basic residue polybasic cleavage site (Appendix 2 Table), characteristic of a highly pathogenic phenotype. We detected amino acid differences in the receptor-binding sites and glycosylation sites of the hemagglutinin (HA) proteins, which may contribute to altered receptor-binding preference among the 9 HPAI/LPAI H5 viruses. Of note, all H5 viruses possessed >1 of the 7 observed HA amino acid mutations previously reported to increase virus binding to α2,6-linked sialic acid receptors: 94N, 133A, 154D, 155N, 156A, 188I, and 189R (*20**–**23*). HPAI H5N6/H5N8 viruses and HPAI H5N1/SZ1 also contained a single or double HA amino acid mutation(s), 218Q and 223R, required for binding fucosylated α2,3-linked sialic acid receptors (*24*). Of the 29 molecular marker positions associated with virulence, transmission, replication efficiency, and adaptation in mammals, which were differentially expressed among the 9 H5 viruses in neuraminidase (NA), PB2, PB1, PB1-F2, polymerase acidic, nucleocapsid, matrix (M) 1, M2, nonstructural (NS) 1 and NS2 proteins (*11*,*17*,*25**–**34*), most (20) molecular markers were found in HPAI H5N1/483 viruses; 8–11 in HPAI H5N1/SZ1 and HPAI H5N6 viruses, except for avHPAI H5N6/18259, which contained only 2, and 4–6 in HPAI/LPAI H5N8 viruses. The well-known mammalian adaptation marker PB2 627K was detected in only 2 HPAI H5 human isolates, HPAI H5N1/483 and HPAI H5N6/39715; PB2 701N was not detected in any of the H5 viruses. The NA 96A and M2 31N mutations, which confer resistance, were observed in avHPAI H5N6/18259 (resistant to zanamivir and oseltamivir) and avHPAI H5N6/MP692 (resistant to amantadine and rimantadine) (*35*, *36*).

### Receptor Binding 

We used untreated and desialylated 0.5% turkey red blood cells (TRBCs) to determine the receptor-binding specificities of the HPAI H5N6/H5N8 avian isolates. Cleavage of α2,3-linked unbranched sialic acid by Glyko Sialidase S (Agilent, https://www.agilent.com) reduced the hemagglutination of avHPAI H5N6/18259 by 16-fold but did not affect hemagglutination of avHPAI H5N6/DK01, avHPAI H5N6/MP692, avHPAI H5N8/636099, and avHPAI H5N8/642613 (Table). On the other hand, cleavage of both α2,3- and α2,6-linked unbranched sialic acid by Glyko Sialidase C (Agilent) resulted in 2- to 16-fold drops in hemagglutination of avHPAI H5N6/DK01, avHPAI H5N6/MP692, avHPAI H5N8/636099, and avHPAI H5N8/642613. However, there was no further reduction in the level of hemagglutination of avHPAI H5N6/18259 in Sialidase C–treated versus Sialidase S–treated TRBCs. These data suggest that avHPAI H5N6/18259 predominantly binds α2,3-linked sialic acid, but the other 4 HPAI H5N6/H5N8 avian isolates can bind to receptors with α2,6 linkage. Treatment of TRBCs with Sialidase S and Sialidase C similarly abolished the hemagglutination of predominantly α2,3-linked sialic acid binders HPAI H5N1/483 and HPAI H5N6/39715 (*17*, *18*) and partially prevented the hemagglutination of HPAI H5N1/SZ1 and avLPAI H5N8/MP5883. The hemagglutination of predominantly α2,6-linked sialic acid binder pH1N1 (*37*) was not affected by Sialidase S but was partly inhibited by Sialidase C. The ability of the tested viruses, apart from HPAI H5N1/483 and HPAI H5N6/39715, to maintain some hemagglutination to Sialidase C–treated TRBCs suggests their potential to bind to receptors other than sialic acid with α2,3 and α2,6 unbranched linkages.

**Table:** Effects of desialylation on influenza A virus hemagglutination of turkey red blood cells*

Virus	0.5% Turkey red blood cells		
	Untreated	Sialidase S†	Sialidase C†
Influenza A(H1N1)pdm09	64	64	8
HPAI H5N1/483	256	0	0
HPAI H5N1/SZ1	64	4	4
HPAI H5N6/39715	128	0	0
avHPAI H5N6/DK01	128	128	32
avHPAI H5N6/18259	64	4	4
avHPAI H5N6/MP692	64	64	32
avHPAI H5N8/636099	128	128	8
avHPAI H5N8/642613	256	256	64
avLPAI H5N8/MP5883	32	4	4

*Hemagglutination titers were calculated as the reciprocal of the highest dilution that gave hemagglutination. Experiments were performed in triplicate and led to identical results. Av, avian; HPAI, highly pathogenic avian influenza. †Agilent (https://www.agilent.com).

## Discussion

We demonstrated that the 5 HPAI H5N6/H5N8 clade 2.3.4.4b, e, and h avian isolates from 2016–2018 replicated productively and to similar competence in human airway organoids and alveolar epithelial cells. Replication was less efficient than that of pH1N1 and HPAI H5N6/39715 in human airway organoids and HPAI H5N1/483 and HPAI H5N6/39715 in alveolar epithelial cells. The isolates showed differential cellular tropism in human airway organoids; some infected all 4 major epithelial cell types, including ciliated cells, club cells, goblet cells, and basal cells, similar to pH1N1 and the HPAI H5 human isolates. Compared with HPAI H5N1/483, HPAI H5N6/H5N8 clade 2.3.4.4 viruses induced fewer proinflammatory cytokines and chemokines.

Tropism of influenza virus for the human conducting airways and lower lung epithelial cells is a useful parameter for assessing its zoonotic and pandemic threat in terms of receptor-binding capacity, and the World Health Organization has listed infection of human bronchus explants as a reliable risk-assessment platform (*38*). In addition, replication in the conducting airways has been suggested as a prerequisite for influenza virus acquisition of efficient human-to-human transmission (*38*, *39*). We have previously demonstrated that tropism and replication competence of influenza viruses in human airway organoids mimicked those found in human bronchus explants (*18*, *19*). However, human airway organoids are more readily available for studies because they can be easily rescued from long-term cryopreservation and expanded.

In this study, the productive but lower replication competence of HPAI H5N6/H5N8 clade 2.3.4.4b, e, and h avian isolates compared with that of pH1N1, HPAI H5N1/483, and HPAI H5N6/39715 in human airway organoids, alveolar epithelial cells, or both, suggests lower zoonotic potential and transmissibility of these avian isolates in humans. Knowing that pH1N1 was a successful pandemic virus that infected an estimated 20%–27% of the world population during the first year of circulation (*40*) and that HPAI subtypes H5N1 and H5N6 are able to cause zoonotic infection but lack efficient human-to-human transmission to cause a pandemic to date, we could extrapolate that the risk for human-to-human transmission of HPAI H5N6/H5N8 clade 2.3.4.4b, e, and h avian isolates in this study is low but that zoonotic infection caused by direct contact with infected poultry or contaminated environment is possible, as in the case of HPAI H5N6/39715 and most previous human cases of HPAI H5N6/H5N8 clade 2.3.4.4 infection (*7*, *8*). Perhaps it would be best to confirm the virus transmissibility in animal models, such as ferrets and guinea pigs. However, the inability of HPAI H5N6/39715 to transmit among ferrets via respiratory droplets (*41*) makes it highly doubtful that the HPAI H5N6/H5N8 clade 2.3.4.4b, e, and h avian isolates in this study would transmit better. It has been shown that replication in human bronchus explants correlates with respiratory droplet transmission of swine influenza viruses in ferrets (*39*). Respiratory droplet transmission of HPAI H5N6/H5N8 clade 2.3.4.4 viruses has not been demonstrated in any animal model examined (*8*,*12*,*13*,*16*,*41*,*42*), which is consistent with their lack of human-to-human spread. However, direct contact transmission of some HPAI H5N6 clade 2.3.4.4 viruses was previously demonstrated in ferrets and guinea pigs (*8*,*12*,*13*,*16*), thus indicating potential risk.

Although transmissibility of the HPAI H5N6/H5N8 clade 2.3.4.4 viruses in humans seems to be low, persons with frequent exposure to poultry and wild birds should be educated to practice precautionary measures because of ongoing outbreaks among avian species. Repeated virus introductions provide a chance for adaptation to enhance virus replication efficiency, virulence, and transmissibility in humans (*14* *43*). An avian influenza HPAI H5N1 virus became airborne transmissible among ferrets after acquiring mutations during passage in ferrets (*43*). Moreover, reassortments of viral gene segments in avian species are continuously associated with the evolution of H5Nx viruses (*1*,*2*,*8*).

The lower replication competence of avHPAI H5N6/18259, avHPAI H5N6/MP692, and avHPAI H5N8/636099 compared with that of HPAI H5N6/39715 in human airway organoids, despite being similarly able to infect all 4 major epithelial cell types, may partially be explained by their lack of the mammalian adaptation marker PB2 627K that is present in HPAI H5N6/39715. PB2 627K enhances polymerase activity and avian virus replication in mammalian cells. Likewise, the presence of PB2 627E instead of 627K and a higher preference for α2,6-linked sialic acid among the 5 HPAI H5N6/H5N8 avian isolates may partially be accounted for by their lower replication competence compared with that of HPAI H5N1/483 and HPAI H5N6/39715 in alveolar epithelial cells, which are predominantly lined with α2,3-linked sialic acid receptors (*44*).

Because human respiratory epithelium is the primary target for influenza viruses, knowledge of the innate host responses in the respiratory epithelium, especially that of the alveolar epithelium, on severe influenza infection, such as infection with HPAI H5N1, helps elucidate the pathogenesis of the dysregulated cytokine responses and severe pneumonia associated with the disease. In this study, HPAI H5N6/H5N8 clade 2.3.4.4 viruses, including the HPAI H5N6/39715 human isolate, were found to be low inducers of proinflammatory cytokines and chemokines in alveolar epithelial cells and in human airway organoids, which is unlikely to contribute to the pathogenesis of severe disease. In connection, we found fewer molecular markers associated with virulence in the tested clade 2.3.4.4 viruses compared with HPAI H5N1/483. Our data are consistent with those from previous studies that reported low cytokine/chemokine induction of HPAI H5N6 clade 2.3.4.4 viruses in alveolar epithelial cells and low virulence of clade 2.3.4.4 HPAI H5N8 viruses in humans, ferrets, and mice (*9*,*10*,*16*,*17*,*42*,*45*,*46*). However, those findings cannot help explain the high fatality rate of HPAI H5N6 clade 2.3.4.4 infection in humans and the high cytokine/chemokine levels in the serum of some patients infected with HPAI H5N6 clade 2.3.4.4, which are comparable to those infected with HPAI H5N1 (*7*). One possible explanation may be the differences in virus strains involved. Some studies have pointed out that HPAI H5N6 clade 2.3.4.4 viruses exhibited pronounced strain-specific heterogeneity regarding their capacity to cause severe and fatal disease (*11*,*13*). Another reason maybe the differences in the patients’ health conditions, including their ages and underlying medical conditions, in naturally infected patients (*47*). Moreover, apart from innate host responses in the epithelium, other factors (e.g., responses from immune cells and viral tropism for deeper body cells/tissues) are likely to contribute to the final outcomes of the viral infection.

Previous studies that used different receptor-binding assays demonstrated both dual receptor-binding preference and preference to α2,3-linked sialic acid of H5 clade 2.3.4.4 viruses (*13*,*16*,*17*,*48*). Although avHPAI H5N6/18259 predominantly bound α2,3-linked sialic acid in our desialylation–hemagglutination assay, it showed comparable productive replication to the 4 α2,6-linked sialic acid–binding HPAI H5N6/H5N8 avian isolates in human airway organoids, which are predominantly lined with α2,6-linked sialic acid receptors (*19*). It also showed tropism for all 4 major epithelial cell types infected by the predominantly α2,6-linked sialic acid binder pH1N1 in human airway organoids. Likewise, Kwon et al. showed efficient replication of 2 HPAI H5N6/H5N8 clade 2.3.4.4 avian viruses with strong preferential binding to α2,3-linked sialic acid in human bronchial epithelial cells (*16*). These data suggest that the α2,3/α2,6 linkage type alone may not be sufficient to successfully determine influenza virus tropism in the human respiratory tract. More comprehensive but focused glycan arrays that express the wide spectrum of relevant glycans in human airways are needed (*37*). Unfortunately, only a few glycans present in currently available glycan arrays are found in the human respiratory tract.

Because our desialylation–hemagglutination assay involved sialidases that cleaved only unbranched α2,3- and α2,6-linked sialic acid, binding of the H5 viruses to branched sialic acid could not be assessed, which might have contributed to the maintenance of hemagglutination titers in Sialidase S–treated and Sialidase C–treated TRBCs. Fucosylated α2,3-linked sialic acid is a potential branched receptor. It was shown to bind H5 HA clade 2.3.4.4 proteins with substitutions in K218Q, S223R, or both, which can be found in our tested HPAI H5N6/H5N8 clade 2.3.4.4 viruses (*24*). Complex glycans containing fucosylated structures with sialylation, including sialyl-Lewis X (NeuAcα2–3Galβ1–4[Fucα1–3]GlcNAc) and sialyl-Lewis A (NeuAcα2–3Galβ1–3[Fucα1–4]GlcNAc), have been detected in human lung tissues and the epithelium of small airways (*49*). Binding to these glycans might increase the infectivity and hence replication of clade 2.3.4.4 viruses in human airways. In contrast, earlier HPAI H5N1 viruses without the double HA amino acid substitutions have reduced binding to glycans that are fucosylated at the penultimate GlcNAc residue (*24*,*50*).

In conclusion, the productive viral replication and tropism for different cell types in human airway organoids and alveolar epithelial cells suggest zoonotic potential of the 5 HPAI H5N6/H5N8 clade 2.3.4.4b, e, and h avian isolates from 2016–2018. However, the risk for human-to-human transmission seems to be low. The low levels of proinflammatory cytokines and chemokines induced by these viruses in the human respiratory epithelium are unlikely to contribute to the pathogenesis of severe disease. However, the persistent circulation of clade 2.3.4.4 viruses among avian populations and periodic infection of human hosts enable ongoing evolution of the viruses with the possibility of acquiring better transmissibility, higher pathogenicity, or both, in humans. Therefore, education, vaccine development, surveillance, and risk assessment surrounding these viruses should continue.

Appendix 1 Supplemental methods for risk assessment for highly pathogenic avian influenza A(H5N6/H5N8) clade 2.3.4.4 viruses.

Appendix 2 Supplemental results for risk assessment for highly pathogenic avian influenza A(H5N6/H5N8) clade 2.3.4.4 viruses.
