# Supplementary material for: Risk Assessment for Highly Pathogenic Avian Influenza A(H5N6/H5N8) Clade 2.3.4.4 Viruses
Source: Emerg Infect Dis. 2021 Oct;27(10):2619–27. doi: 10.3201/eid2710.210297 (PMC8462306; doi:10.3201/eid2710.210297)
Supplement: Appendix 1 — Supplemental methods for risk assessment for highly pathogenic avian influenza A(H5N6/H5N8) clade 2.3.4.4 viruses. [file 21-0297-Techapp-s1.pdf]

Article DOI: https://doi.org/10.3201/eid2710.210297

Risk Assessment for Highly Pathogenic Avian Influenza A(H5N6/H5N8) Clade 2.3.4.4 Viruses

Appendix 1

Methods

50% Tissue Culture Infectious Dose ($TCID_{50}$) assay

Confluent 96-well MDCK cell plates were seeded one day before. Cells were then washed with 1x phosphate buffered saline (PBS), pH 7.4 without Ca2+ or Mg2+, and filled with new medium: MEM (Gibco) supplemented with 100 U/ml Penicillin-Streptomycin (Gibco), 0.025 M HEPES (Gibco), and 1 µg/ml TPCK-treated trypsin from bovine pancreas (Sigma-Aldrich). Virus was serial diluted at half-log_{10} dilution and added to the cell plates in quadruplicates. Plates were incubated in a humidified incubator at 37°C and 5% CO_2 until 72 hours post infection (hpi). Karber method was used to determine the viral dilution endpoints leading to cytopathic effect (CPE) in 50% of inoculated wells.

Human lung tissues

Human lung tissues were removed from patients undergoing surgical resection as part of a routine clinical care in Hong Kong. After fulfilling all diagnostic requirements, fresh non-tumour residual lung tissue was used for this study. Informed consent was obtained from patients and approval was granted by the Institutional Review Board of the University of Hong Kong and the Hospital Authority (Hong Kong West) (approval no: UW 18-196).

Human airway organoids – Isolation and Culture

Human lung tissues were minced and washed with AdDF+++ basic medium, which consisted of Advanced DMEM/F-12 (Gibco) supplemented with 1x GlutaMAX™ (Gibco), 10 mM HEPES (Gibco), and 100 U/ml Penicillin-Streptomycin (Gibco). Tissues were digested with 2 mg/ml collagenase type II (Sigma-Aldrich) in a 37°C shaker for 1 h. 2% fetal bovine serum (FBS) was added to stop the digestion. The tissue suspension was added with 100 µg/ml DNase I

(Sigma-Aldrich), sheared with plastic pipettes, and strained with 100 μm and 70 μm filters. The collected filtrate was centrifuged at 600 × g for 5 min at 4ºC. Supernatant was removed. The cell pellet was resuspended in red blood cell lysis buffer (Roche) and incubated at room temperature for 5 min, followed by the addition of AdDF+++ basic medium and centrifugation at 600 × g for 5 min at 4ºC. 10 mg/ml cold Cultrex growth factor reduced basement membrane extract type 2 Matrigel (Trevigen) was added to the cell pellet and 40 μl droplets of the cell-in-Matrigel suspension was aliquoted to prewarmed 24-well suspension culture plates. The plates were incubated at 37ºC for 15-30 min. 1 ml organoid medium was added to each well as previously described (*1*,*2*). The plates were incubated in a humidified incubator at 37ºC and 5% CO_2. Medium was changed every 4 days and organoids were passaged every 2 weeks. To passage, organoids-in-Matrigel droplets were resuspended in cold AdDF+++, sheared with flamed glass Pasteur pipettes, and centrifuged at 400 × g for 5 min at 4ºC. Organoid fragments were re-embedded in new cold Matrigel with the usual expansion of 6 times the original Matrigel droplet number. After 14 days in culture at 37ºC and 5% CO_2, human airway organoids were ready for infection.

Human airway organoids – Infection

Human airway organoids of approximately 100 μm in diameter were removed from Matrigel droplets and slightly broken open by shearing with flamed glass Pasteur pipettes. Around 100–200 organoids were infected with each virus at 6 log $TCID_{50}$/ml for 1 h at 37ºC. Organoids were washed 3 times: twice with AdDF+++ basic medium and once with organoid medium. They were re-embedded in Matrigel and cultured in organoid medium in a humidified incubator at 37ºC and 5% CO_2. Culture supernatant was collected at 1, 24, 48 and 72 hpi for virus titration by $TCID_{50}$ assay. Organoids were fixed in 4% paraformaldehyde at 24 and 48 hpi for immunohistochemical double staining. Organoid lysates were collected at 24 hpi by adding Buffer RLT (Qiagen) supplemented with 1% β-mercaptoethanol. Experiments were performed with organoids isolated from at least 4 different donors.

Primary human alveolar epithelial cells – Isolation and Culture

Human lung tissues were minced and washed 3 times using HBSS (Gibco) supplemented with 0.7 mM sodium bicarbonate (Gibco) at pH 7.4. Tissues were digested using 0.5% trypsin (Gibco) and 4 U/ml elastase (Worthington Biochemical Corporation) in a 37ºC water bath for 40 min. DMEM/F12 medium (Gibco) supplemented with 40% FBS and 350 U/ml DNase I

(Sigma-Aldrich) were added to stop the digestion. The tissue suspension was pipetted up and down with 10 ml plastic pipettes for 10 min and strained through 50 μm filters. The filtrate was centrifuged at 1500 rpm for 5 min. Supernatant was removed and the cell pellet was resuspended in a 1:1 mixture of DMEM/F12 medium and small airway growth medium (SAGM) (Lonza) supplemented with 5% FBS and 350 U/ml DNase I. The cell suspension was seeded to tissue culture flasks (Corning) and incubated in a humidified incubator at 37ºC and 5% CO_2 for 90 min. Cells not adhered to the flasks were collected and centrifuged at 1500 rpm for 5 min. Supernatant was removed. The cell pellet resuspended in SAGM supplemented with 100 U/ml Penicillin-Streptomycin (Gibco) was seeded to new tissue culture flasks. Cells were kept in a humidified incubator at 37ºC and 5% CO_2. After 60 h, culture medium was changed every day until reaching 75% confluence. Cells were then trypsinized and seeded for infection.

Primary human alveolar epithelial cells – Infection

1×10^5 cells/well were seeded in 24-well tissue culture plates a day before. Attached cells were washed 3 times with 1x PBS, pH 7.4 without Ca2+ or Mg2+, and infected at multiplicities of infection (MOIs) of 0.01 and 2 for 1 h at 37ºC and 5% CO_2. The infected cells were washed 3 times with 1x PBS and replenished with 1 ml SAGM (Lonza) supplemented with 100 U/ml Penicillin-Streptomycin (Gibco) and 0.25 μg/ml TPCK-treated trypsin from bovine pancreas (Sigma-Aldrich). They were cultured in a humidified incubator at 37ºC and 5% CO_2. Culture supernatant was collected at 1, 24, 48, and 72 hpi for virus titration by $TCID_{50}$ assay. Cell lysates were collected at 24 hpi by adding 350 μl Buffer RLT (Qiagen) with 3.5 μl β-mercaptoethanol to each well. Experiments were performed with cells isolated from 3 different donors.

Immunohistochemistry double staining

The staining procedures were as previously described with slight modifications (*1*,*2*). Infected organoids were washed with cold AdDF+++ and removed from Matrigel droplets pior to being fixed in 4% paraformaldehyde. Fixed tissues were embedded in paraffin. After sectioning and deparaffinization, the sections were incubated with 0.05% Pronase (Roche) at 37ºC for 16 min and 3% H_2O_2 at room temperature for 10 min. Subsequently, ImmPRESS® HRP Anti-Mouse IgG (Peroxidase) Polymer Detection Kit, made in Horse (Vector Laboratories) were used for staining with the first antibody according to the manufacturer's instructions. Briefly, sections were blocked with 2.5% horse serum. Anti-influenza A virus (IAV) nucleoprotein-specific mouse monoclonal antibody HB65 (European Veterinary Laboratory) was

added to the sections at a dilution of 1/10 and incubated at room temperature for 1 h. After a brief wash, the ImmPRESS Reagent was added to the sections and incubated for 1 h at room temperature. Sections were then rinsed, and the slides were developed with 0.5 mg/ml DAB (Vector Laboratories) for 2-3 min or VectorRed (Vector Laboratories) for 10 min. For staining with the second antibody, the sections were first microwaved at 95ºC for 15 min in 10 mM citrate buffer, pH 6.0. ImmPRESS®-AP Anti-Mouse IgG (alkaline phosphatase) Polymer Detection Kit (Vector Laboratories) was used with 1/1000 diluted anti-acetylated α tubulin (Santa Cruz) antibody and 1/50 diluted anti-MUC5AC (ThermoFisher) antibody while ImmPRESS®-AP Anti-Rabbit IgG (alkaline phosphatase) Polymer Detection Kit (Vector Laboratories) was used with 1/100 diluted anti-SCGB1A1/CC10 (Protein-tech) antibody and 1/100 diluted anti-p63-α (Cell Signaling Technology) antibody according to the manufacturer's instructions. Incubation with the cell-type specific marker antibodies and the ImmPRESS Reagents took 60-90 min and 60 min, respectively, at room temperature. The sections were developed using VectorRed (Vector Laboratories) or DAB for 3 min. The nuclei were counterstained with Mayer's Haematoxylin for 10 seconds and the sections were blued with Scott's tap water, air dried, and mounted with Permount (Fisher Scientific).

Cytokine and chemokine expression

RNA was extracted from cell lysates using MiniBEST universal RNA extraction kit (Takara) and reverse transcribed using PrimerScript RT reagent Kit (Takara). mRNA expression was quantified using real-time PCR amplification with SYBR *Premix Ex* Taq II (Tli RNase H Plus) (Takara) and an ABI ViiA™ 7 real-time PCR system (Applied Biosystems). The procedures were performed according to the manufacturers' instructions. Absolute mRNA copy numbers of β-actin (forward primer: 5'-TGGATCAGCAAGCAGGAGTATG-3' and reverse primer: 5'-GCATTTGCGGTGGACGAT-3'), IAV matrix (M) gene (forward primer: 5'-GGCATTTTGGACAAAKCGTCTA-3' and reverse primer: 5'-CTTCTAACCGAGGTCGAAACG-3'), interferon-β (IFNβ) (forward primer: 5'-CAACTTGCTTGGATTCCTACAAAG-3' and reverse primer: 5'-TGCCACAGGAGCTTCTGACA-3'), interferon-λ1 (IFN-λ1) (forward primer: 5'-GCCCCCAAAAAGGAGTCCG-3' and reverse primer: 5'-AGGTTCCCATCGGCCACATA-3'), C-C motif chemokine ligand 5 (CCL5) (forward primer: 5'-CTTTGCCAGGGCTCTGTGA-3' and reverse primer: 5'-GCAGTGTTCCTCCCCTCCTT-3'), C-X-C motif chemokine 10

(CXCL10) (forward primer: 5’-ATTATTCCTGCAAGCCAATTTTG-3’ and reverse primer: 5’-TCACCCTTCTTTTTCATTGTAGCA-3’), tumor necrosis factor α (TNFα) (forward primer: 5’-GCAGGTCTACTTTGGGATCATTG-3’ and reverse primer: 5’-GCGTTTGGGAAGGTTGGA-3’), interleukin 6 (IL-6) (forward primer: 5’-GCATGGGCACCTCAGATTGT-3’ and reverse primer: 5’-TGCCCAGTGGACAGGTTTCT-3’), interferon-stimulated gene 15 (ISG15) (forward primer: 5’-CAAATGCGACGAACCTCTGA-3’ and reverse primer: 5’-CCGCTCACTTGCTGCTTCA-3’), and interferon-induced GTP-binding protein Mx1 (MX1) (forward primer: 5’-GAGGCCAGCAAGCGCAT-3’ and reverse primer: 5’-TGGAGCATGAAGAACTGGATGA-3’) were determined with standard curve method (*2*,*3*). mRNA expression of all genes was normalized to that of β-actin.

Desialylation–haemagglutination assay

The effect of desialylation on IAV haemagglutination of Turkey red blood cells (TRBCs) was studied as previously described (*2*,*3*). TRBCs (Lampire) in Alsevers were washed and diluted to 0.5% in 1x PBS, pH 7.4 without Ca2+ or Mg2+. They were treated with either 20 mU/ml Glyko® Sialidase S™ (Prozyme) or 200 mU/ml Glyko® Sialidase C™ (Aglilent) for 2 h at 37°C. After the treatment, TRBCs were washed with 1x PBS, centrifuged at 2000 rpm for 5 min, and resuspended in 1x PBS to 0.5%. Two-fold serial dilutions of viruses were carried out with 1x PBS in Greiner CELLSTAR® Clear V-bottom 96-well plates (Sigma-Aldrich) in triplicates. 50 µl of the treated or untreated 0.5% TRBCs were added to 50 µl of the diluted viruses/1x PBS (negative control) in each well. The plates were incubated at room temperature for 20 min. The haemagglutination titres were calculated as the reciprocal of the highest dilution that gave haemagglutination.

Phylogeny analysis

Clade classification was performed by comparative phylogenetic analysis. Seventy-one selected representative highly pathogenic avian influenza (HPAI) H5 HA sequences in Global Initiative on Sharing All Influenza Data (GISAID) and GenBank between 2007 and 2021 (*4*) (Appendix 1 Table) were used to build a phylogenetic tree based on the nucleotide sequences coding for the mature HA1 protein in this study. Multiple sequence alignment was performed using the MUSCLE algorithm. Phylogenetic analysis was inferred by using the maximum likelihood method and the Tamura-Nei model in MEGA version X. Stability of the phylogenetic tree branch topology was tested using 1000 bootstrap replicates.

Statistical and sequencing analysis

Area Under Curve (AUC) and statistical analyses were performed using GraphPad Prism, version 8.4.3. AUC values were calculated as the areas under the replication kinetic curves from 1 or 24 to 72 hpi above the $TCID_{50}$ assay detection limit (1.5 log $TCID_{50}$/ml) using the trapezoid rule (*5*). AUC values and mRNA levels were compared by one-way analysis of variance (one-way ANOVA) with *Bonferroni* post-tests. Viral titres at each time point post-infection were compared by two-way ANOVA with *Bonferroni* post-tests. Sequence alignment and analyses were conducted using MEGA version X.

References

1. Hui KPY, Ching RHH, Chan SKH, Nicholls JM, Sachs N, Clevers H, et al. Tropism, replication competence, and innate immune responses of influenza virus: an analysis of human airway organoids and ex-vivo bronchus cultures. Lancet Respir Med. 2018;6:846–54. PubMed https://doi.org/10.1016/S2213-2600(18)30236-4
2. Bui CHT, Chan RWY, Ng MMT, Cheung MC, Ng KC, Chan MPK, et al. Tropism of influenza B viruses in human respiratory tract explants and airway organoids. Eur Respir J. 2019;54:1900008. PubMed https://doi.org/10.1183/13993003.00008-2019
3. Hui KP, Chan LL, Kuok DI, Mok CK, Yang ZF, Li RF, et al. Tropism and innate host responses of influenza A/H5N6 virus: an analysis of *ex vivo* and *in vitro* cultures of the human respiratory tract. Eur Respir J. 2017;49:1601710. PubMed https://doi.org/10.1183/13993003.01710-2016
4. World Health Organization. Antigenic and genetic characteristics of zoonotic influenza viruses and candidate vaccine viruses developed for potential use in human vaccines - March 2021 [cited 2021 May 24]. https://www.who.int/influenza/vaccines/virus/202103_zoonotic_vaccinevirusupdate.pdf?ua=1
5. GraphPad Software. Area under the curve [cited 2021 Jun 7]. https://www.graphpad.com/guides/prism/latest/statistics/stat_area_under_the_curve.htm

Appendix 1Table. Highly pathogenic avian influenza (HPAI) H5 strains isolated between 2007 and 2021 and their HA sequence accession numbers in Global Initiative on Sharing All Influenza Data (GISAID) and GenBank

Clades	Reference strains*	Accession no.
2.3.4.4a	A/chicken/Sichuan/J1/2014	EPI675770
	A/Sichuan/26221/2014	EPI533583
	A/chicken/Vietnam/NCVD14-A324/2014	EPI894943
2.3.4.4b	A/wild duck/Korea/H331/2020 (N8)	EPI1846698
	A/chicken/Astrakhan/321-05/2020 (N8)	EPI1846985
	A/Astrakhan/3212/2020 (N8)	EPI1846961
	A/turkey/Rostov-on-Don/332-08/2021 (N8)	EPI1848742
	A/buzzard/Germany-MV/AI02166/2020 (N5)	EPI1811564
	A/Gallus_gallus/Belgium/12168_002/2020 (N5)	EPI1814627
	A/peregrine_falcon/Ireland/20VIR7872-1/2020 (N8)	EPI1841769
	A/barnacle goose/Denmark/14138-1/2020-11-04 (N8)	EPI1813753
	A/chicken/Krasnodar/334-02/2021 (N8)	EPI1848790
	A/domestic_goose/Kazakhstan/1-261_2-20-B/2020 (N8)	EPI1811592
	A/goose/Omsk/0002/2020 (N8)	EPI1813121
	A/Eurasian_wigeon/Italy/20VIR7301-362/2020 (N8)	EPI1843650
	A/chicken/Iraq/1/2020 (N8)	EPI1811628
	A/chicken/Rostov-on-Don/766/2018 (N8)	EPI1270931
	A/chicken/Sergiyev Posad/38/2017 (N8)	EPI961459
	A/mute swan/Shimane/3211A001/2017	EPI1127538
	A/Eurasian_Wigeon/Netherlands/1/2016 (N8)	EPI1023576
	A/chicken/Egypt/N13732A/2017	MH998393
	A/duck/India/10CA01/2016 (N8)	EPI858836
	A/Fujian-Sanyuan/21099/2017	EPI1202729
	A/environment/Kamchatka/18/2016 (N5)	EPI961478
	A/duck/Bangladesh/19D851/2017	EPI1329121
	A/great crested grebe/Tyva/34/2016 (N8)	EPI823460
	A/northern pintail/Hokkaido/M13/2020 (N8)	EPI1818402
	A/chicken/Czech Republic/1175-1/2020 (N8)	EPI1667598
	A/steamer duck/Germany-SN/AI00346/2020 (N8)	EPI1721676
	A/chicken/Kostroma/1718/2017 (N2)	EPI1158108
2.3.4.4c	A/gyrfalcon/Washington/41088-6/2014 (N8)	EPI569390
	A/American green-winged teal/Washington/195750/2014 (N1)	EPI573286
	A/wigeon/Sakha/1/2014 (N8)	EPI553349
	A/mallard/Korea/W452/2014 (N8)	EPI542628
	A/duck/Beijing/FS01/2014 (N8)	EPI543002
2.3.4.4d	A/oriental magpie robin/HK/6154/2015	EPI1060783
	A/Shenzhen/1/2016	EPI687704
	A/Anhui/33162/2016	EPI1098355
	A/Hubei/29578/2016	EPI961183
2.3.4.4e	A/duck/Taiwan/1702004/2017	EPI915870
	A/duck/Hyogo/1/2016	EPI866708
	A/chicken/Korea/HN1/2016	EPI866085
	A/environment/Fujian/28686/2016	EPI1333910
2.3.4.4f	A/chicken/Vietnam/NCVD-15A59/2015	EPI895070
	A/Whooper Swan/Hunan/4/2016	EPI1639951
2.3.4.4g	A/chicken/Vietnam/Raho4-Cd-20-421/2020	EPI1853938
	A/chicken/Dong Nai/25437VTC/2019	EPI1665384
	A/Hunan/55555/2016	EPI873668
	A/Guangxi/55726/2016	EPI873669
	A/chicken/Japan/AQ-HE144/2015	EPI946761
	A/pigeon/Sichuan/NCXN29/2014 (N1)	EPI590898
	A/muscovy duck/Vietnam/LBM631/2014 (N1)	AB979455
2.3.4.4h	A/common gull/Saratov/1676/2018	EPI1355418
	A/chicken/Nghe An/27VTC/2018	EPI1583716
	A/Guangxi/32797/2018	EPI1352829
	A/Guangdong/18SF020/2018	EPI1352813
	A/Anhui/2021-00011/2020	EPI1848299
	A/Chongqing/00013/2021	EPI1848291
	A/Whooper swan/Xinjiang/1/2020	EPI1718942
	A/Guangxi/13486/2017	EPI1352861
Others	A/chicken/China/AH/2012 (N1)	EPI499478
	A/chicken/Laos/LPQ001/2014	EPI550752
	A/goose/Eastern China/L1214/2012 (N8)	EPI703597
	A/duck/Jiangsu/m234/2012 (N2)	EPI399960
	A/goose/Zhejiang/925104/2014 (N8)	EPI681300
	A/breeder duck/Korea/Gochang1/2014 (N8)	EPI509698
	A/duck/Hebei/3/2011 (N2)	EPI431456

Clades	Reference strains*	Accession no.
	A/duck/Guangdong/wy11/2008 (N5)	EPI493817
	A/goose/Shandong/k1204/2009 (N5)	EPI442001
	A/duck/Guangdong/wy24/2008 (N5)	EPI493833
	A/duck/Laos/XBY118/2015	EPI1850997

*NA subtypes other than N6 are indicated in brackets.

Comparator virus	Reference virus: H1N1 pdm	HPAI H5N1/ 483	HPAI H5N1/ SZ1	HPAI H5N6/ 39715	avHPAI H5N6/ DK01	avHPAI H5N6/ 18259	avHPAI H5N6/ MP692	avHPAI H5N8/ 636099	avHPAI H5N8/ 642613	avLPAI H5N8/ MP5883
**24 hpi - human airway organoids**										
pH1N1										
HPAI H5N1/483	> (*)									
HPAI H5N1/SZ1	> (****)	ns								
HPAI H5N6/39715	ns	ns	ns							
avHPAI H5N6/DK01	> (****)	> (**)	ns	> (****)						
avHPAI H5N6/18259	> (****)	ns	ns	> (**)	ns					
avHPAI H5N6/MP692	> (****)	> (*)	ns	> (***)	ns	ns				
avHPAI H5N8/636099	> (****)	> (***)	ns	> (****)	ns	ns	ns			
avHPAI H5N8/642613	> (****)	> (***)	ns	> (****)	ns	ns	ns	ns		
avLPAI H5N8/MP5883	> (****)	> (**)	ns	> (***)	ns	ns	ns	ns	ns	
**48 hpi - human airway organoids**										
pH1N1										
HPAI H5N1/483	> (**)									
HPAI H5N1/SZ1	> (***)	ns								
HPAI H5N6/39715	ns	ns	< (*)							
avHPAI H5N6/DK01	> (****)	> (***)	> (**)	> (****)						
avHPAI H5N6/18259	> (****)	ns	ns	> (**)	ns					
avHPAI H5N6/MP692	> (****)	ns	ns	> (****)	ns	ns				
avHPAI H5N8/636099	> (****)	> (***)	> (***)	> (****)	ns	ns	ns			
avHPAI H5N8/642613	> (****)	> (****)	> (****)	> (****)	ns	> (*)	ns	ns		
avLPAI H5N8/MP5883	> (****)	> (***)	> (***)	> (****)	ns	> (*)	ns	ns	ns	
**72 hpi - human airway organoids**										
pH1N1										
HPAI H5N1/483	> (****)									
HPAI H5N1/SZ1	> (**)	ns								
HPAI H5N6/39715	ns	< (**)	ns							
avHPAI H5N6/DK01	> (****)	ns	ns	> (****)						
avHPAI H5N6/18259	> (**)	ns	ns	ns	ns					
avHPAI H5N6/MP692	> (****)	ns	ns	> (**)	ns	ns				
avHPAI H5N8/636099	> (****)	ns	ns	> (****)	ns	ns	ns			
avHPAI H5N8/642613	> (****)	ns	> (*)	> (****)	ns	ns	ns	ns		
avLPAI H5N8/MP5883	> (****)	ns	> (*)	> (****)	ns	ns	ns	ns	ns	

Appendix 1 Figure 1. Statistical significance between viral titres at each time point post-infection in A) human airway organoids (Figure, panel A) and B) AECs (Figure, panel B) was calculated by two-way ANOVA with *Bonferroni* post-tests. No statistically significant differences were observed between the viral titres at 1 hour post infection (hpi). *: p≤0.05, **: p≤0.01, ***: p≤0.001, ****: p≤0.0001; >: reference virus value is higher than that of the comparator virus; <: reference virus value is lower than that of the comparator virus; ns: nonsignificant.

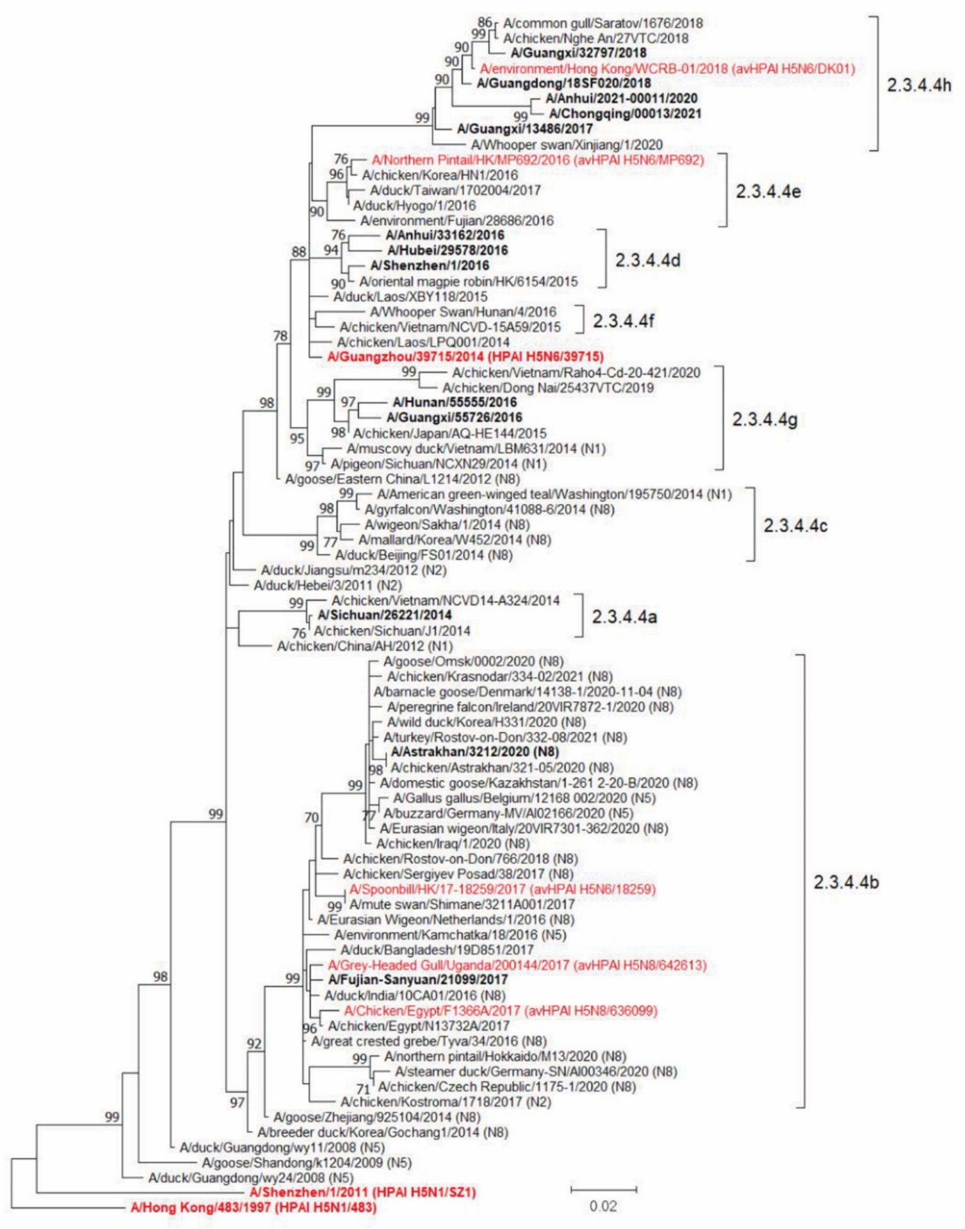

Appendix 1 Figure 2. Phylogenetic relationships of HA genes of A(H5) highly pathogenic avian influenza (HPAI) viruses. The nucleotide sequences coding for the mature HA1 protein were used for analysis. A maximum-likelihood tree using the A(H5) genes of the 8 HPAI viruses in this study (in red) and 71 representative A(H5) HA genes (*4*) (Appendix 1 Table) rooted to A/Hong Kong/483/1997 was constructed using MEGA version X with 1000 replicates. The scale bar represents the number of substitutions per site. Bootstrap values of ≥ 70% are shown. Human viruses are in bold font. NA subtypes other than N6 and virus strain abbreviations used in this study are indicated in brackets.
