# Supplementary material for: Risk Assessment for Highly Pathogenic Avian Influenza A(H5N6/H5N8) Clade 2.3.4.4 Viruses
Source: Emerg Infect Dis. 2021 Oct;27(10):2619–27. doi: 10.3201/eid2710.210297 (PMC8462306; doi:10.3201/eid2710.210297)
Supplement: Appendix 2 — Supplemental results for risk assessment for highly pathogenic avian influenza A(H5N6/H5N8) clade 2.3.4.4 viruses. [file 21-0297-Techapp-s2.pdf]

Article DOI: https://doi.org/10.3201/eid2710.210297

Risk Assessment for Highly Pathogenic Avian Influenza A(H5N6/H5N8) Clade 2.3.4.4 Viruses

Appendix 2

Appendix 2 Table. Molecular features associated with virus receptor-binding preference, mammalian adaptation, pathogenicity, replication efficiency, transmission, and antiviral drug resistance in risk assessment for HPAI influenza A(H5Nx) clade 2.3.4.4 viruses*

		HA†																NA‡
					Receptor binding site													
		Cleavage site	Glycosylation site		130-loop						190-helix					220-loop		Stalk deletion
Viruses	Clade	323–331	154–156	94	126	129	133	141	151	152	183	185	188	189	192	218	223	(aa)
HPAI H5N1/483	0	RE**RRRKKR**↓G	NST	**N**	D	S	S	S	I	K	D	A	T	K	Q	K	S	**Yes (50–68)**
HPAI H5N1/SZ1	2.3.2.1b	IE**RRRRKR**↓G	**DDA**	S	E	L	**A**	S	L	K	D	A	**I**	**R**	K	K	**R**	**Yes (49–68)**
HPAI H5N6/39715	2.3.4.4	RE**RRRKR**↓G	NDT	**N**	E	L	**A**	P	T	K	N	A	T	N	K	**Q**	**R**	**Yes (58–68)**
avHPAI H5N6/DK01	2.3.4.4h	RE**RRRKR**↓G	ND**A**	**N**		S	**A**	A	T	K	S	E	A	D	K	**Q**	**R**	**Yes (58–68)**
avHPAI H5N6/18259	2.3.4.4b	RE**RRRKR**↓G	ND**A**	S	E	L	**A**	P	I	N	N	E	T	N	K	**Q**	**R**	No
avHPAI H5N6/MP692	2.3.4.4e	RE**RRRRKR**↓G	ND**A**	**N**	E	L	S	P	T	K	N	A	T	N	K	**Q**	Q	**Yes (58–68)**
avHPAI H5N8/636099	2.3.4.4b	RE**KRRKR**↓G	NDE	S	E	L	**A**	P	I	K	N	E	T	N	K	**Q**	**R**	No
avHPAI H5N8/642613	2.3.4.4b	RE**KRRKR**↓G	ND**A**	S	E	L	**A**	P	I	K	N	E	T	N	K	**Q**	**R**	No
avLPAI H5N8/MP5883	NA	RET**R**↓G	N**NA**	D	D	S	S	S	I	K	D	A	T	K	Q	K	S	No
		Pathogenicity		Receptor binding preference														

	PB2†							PB1†	PB1-F2†		PA†			NP†		M1†	M2†		NS1‡					NS2‡		NA†	M2†
																							PDZ ligand domain				
									Total aa										80–84 deletion				227–				
Viruses	64	318	355	627	661	667	701	317	length	66	127	336	404	136	319	15	16	55	n	92	103	106	189	230	31	56	96	31
HPAI H5N1/483	M	**K**	**K**	**K**	**T**	**I**	D	**I**	**90**	**S**	**I**	**M**	A	**M**	N	**I**	**G**	**F**	NO	**E**	L	I	**N**	**EPEV**	**I**	**Y**	V	S
HPAI H5N1/SZ1	**I**	R	R	E	A	V	D	M	57		V	L	**S**	L	N	**I**	E	L	**YES**	**E**	**F**	**M**	D	**ESEV**	M	H	V	S
HPAI H5N6/39715	**I**	R	**K**	**K**	A	V	D	M	11		V	L	**S**	L	N	**I**	E	L	**YES**	**E**	**F**	**M**	D	**ESEV**	M	H	V	S
avHPAI H5N6/DK01	M	R	R	E	S	V	D	M	11		V	L	**S**	L	N	**I**	E	L	**YES**	**E**	**F**	**M**	D	**ESEV**	M	H	V	S
avHPAI H5N6/18259	M	R	R	E	A	V	D	M	11		V	L	A	L	N	V	E	L	NO	D	**F**	**M**	D		M	H	**A**	S
avHPAI H5N6/MP692	M	R	R	E	S	V	D	M	**90**	N	V	L	A	L	N	**I**	E	L	**YES**	**E**	**F**	**M**	D	**ESEV**	M	H	V	**N**
avHPAI H5N8/636099	M	R	R	E	A	V	D	M	**90**	**S**	V	L	A	L	N	V	E	L	NO	D	**F**	**M**	D	**GSEV**	M	H	V	S
avHPAI H5N8/642613	M	R	R	E	A	**I**	D	M	11		V	L	A	L	**K**	**I**	E	L	NO	D	**F**	**M**	D	**GSEV**	M	H	V	S
avLPAI H5N8/MP5883	M	R	R	E	A	V	D	M	**90**	N	V	L	A	L	N	V	E	L	NO	D	**F**	**M**	D	**ESEV**	M	H	V	S
	Virulence, transmission, replication efficiency, and adaptation in mammals																									Antiviral drug resistance	

*Boldface indicates previously identified mutations/molecular markers associated with increased viral phenotypic characteristics (bottom row): pathogenicity; binding to α2,6-linked or fucosylated α2,3-linked sialic acid receptors; virulence, transmission, replication efficiency, and adaptation in mammals; and resistance to zanamivir, oseltamivir, amantadine, and rimantadine. ↓ indicates cleavage position. Blank space indicates deleted/missing amino acid(s). Clade classification was done using the Highly Pathogenic H5N1 Clade Classification Tool and according to the phylogenetic relationships of the mature HA1 protein nucleotide sequences (Appendix 1 Figure 2, https://wwwnc.cdc.gov/EID/article/27/10/21-0297-App1.pdf). The numbering of the amino acid is relative to A/Vietnam/1203/2004† and A/goose/Guangdong/1/1996‡. Numbering of HA is based on mature sequences without the N-terminal signal peptides. Aa, amino acid; HA, hemagglutinin; HPAI, highly pathogenic avian influenza; LPAI, low pathogenicity avian influenza; NA, not applicable.

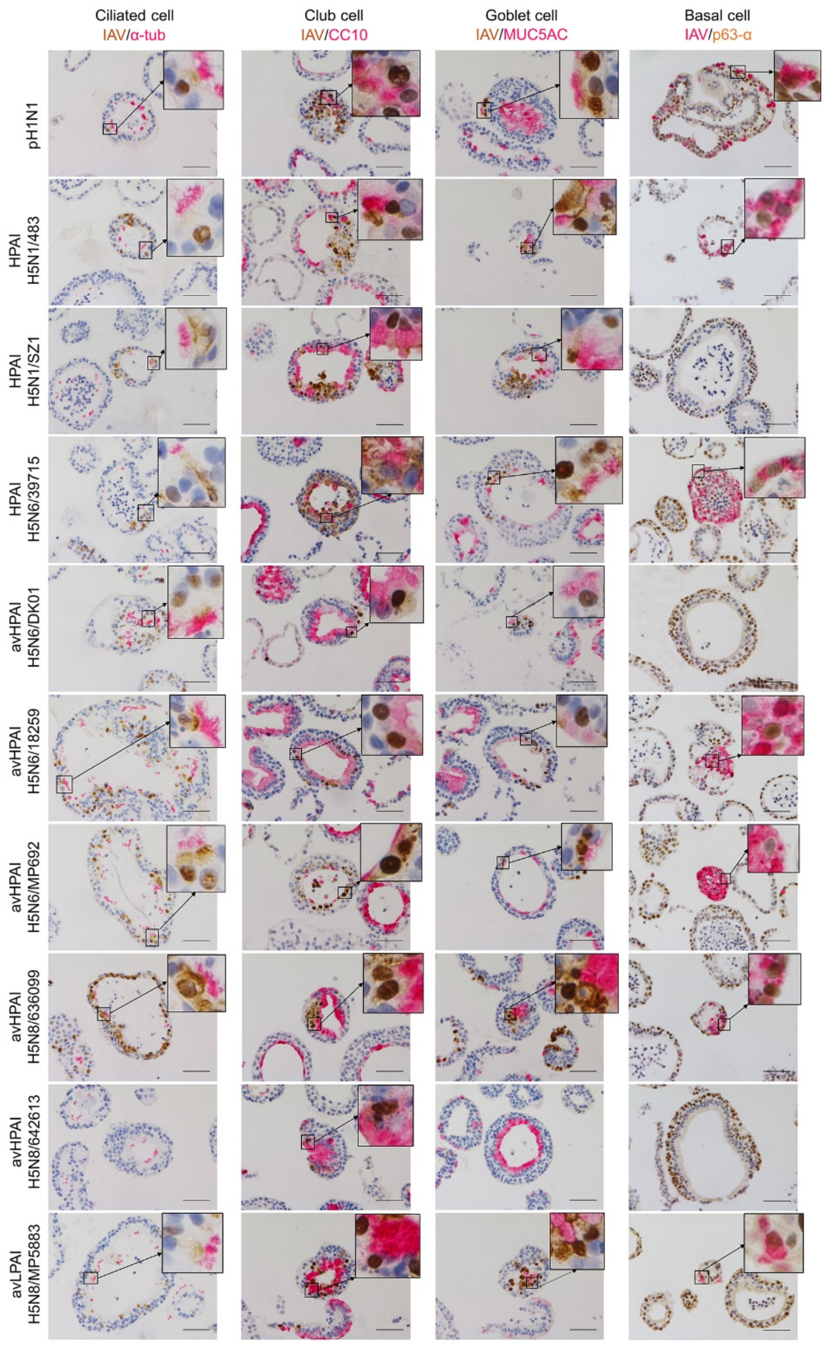

Appendix 2 Figure 1. Representative images of immunohistochemical double staining of influenza A virus (IAV)-infected human airway organoids at 24 or 48 hours post infection (hpi) (n ≥ 3). Ciliated cells, club cells, and goblet cells were co-stained for IAV nucleoprotein (brown) and acetyl-α-tubulin (α-tub) (pink), SCGB1A1/CC10 (CC10) (pink), and MUC5AC (pink), respectively. Basal cells were co-stained for p63-α (brown) and IAV nucleoprotein (pink). Co-localization regions were enlarged in the upper-right corner. Scale bars = 50 μm.

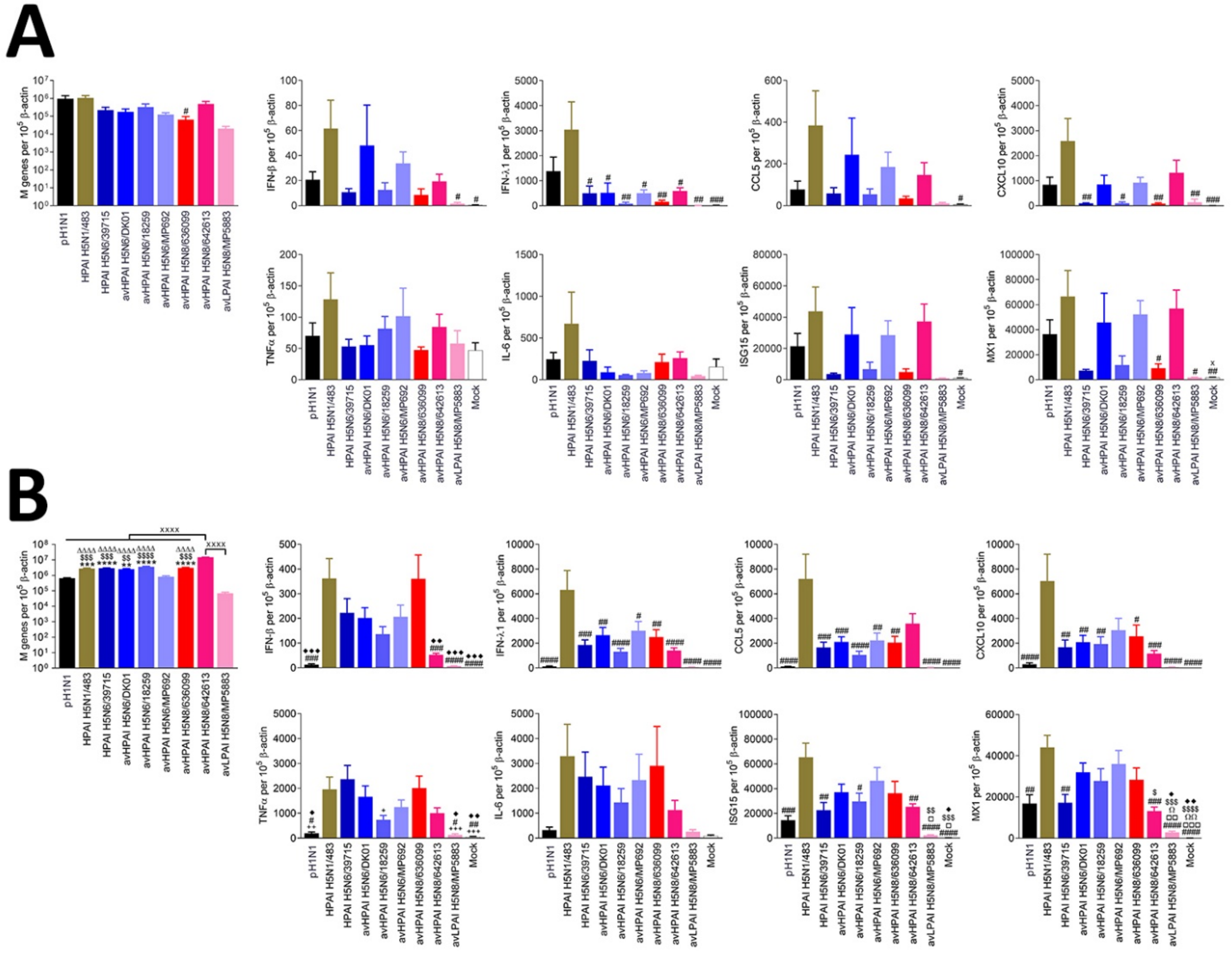

Appendix 2 Figure 2. Cytokine and chemokine mRNA expression in A) human airway organoids infected with 6 log $TCID_{50}$/ml virus and B) primary human alveolar epithelial cells infected at MOI 2 at 37°C at 24 hours post infection (hpi) (mean±SEM, n ≥ 3). mRNA copy numbers of influenza A virus matrix (M) gene, interferon-β (IFN-β), interferon-λ1 (IFN-λ1), C-C motif chemokine ligand 5 (CCL5), C-X-C motif chemokine 10 (CXCL10), tumor necrosis factor α (TNFα), interleukin 6 (IL-6), interferon-stimulated gene 15 (ISG15), and interferon-induced GTP binding protein Mx1 (MX1) were expressed as per 10^5 β-actin copies. Statistical significance between mRNA levels was calculated by one-way ANOVA with *Bonferroni* post-tests. **: p ≤ 0.01, ***: p ≤ 0.001, ****: p ≤ 0.0001 (compared to H1N1pdm); $^{\#}$: p ≤ 0.05, $^{\#\#}$: p ≤ 0.01, $^{\#\#\#}$: p ≤ 0.001, $^{\#\#\#\#}$: p ≤ 0.0001 (compared to HPAI H5N1/483); $^{+}$: p ≤ 0.05, $^{++}$: p ≤ 0.01, $^{+++}$: p ≤ 0.001 (compared to HPAI H5N6/39715); $^{\square}$: p ≤ 0.05, $^{\square\square}$: p ≤ 0.01, $^{\square\square\square}$: p ≤ 0.001 (compared to avHPAI H5N6/DK01); $^{\Omega}$: p ≤ 0.05, $^{\Omega\,\Omega}$: p ≤ 0.01 (compared to avHPAI H5N6/18259); $^{\$}$: p ≤ 0.05, $^{\$\$}$: p ≤ 0.01, $^{\$\$\$}$: p ≤ 0.001, $^{\$\$\$\$}$: p ≤ 0.0001 (compared to avHPAI H5N6/MP692); $^{\blacklozenge}$: p ≤ 0.05, $^{\blacklozenge\blacklozenge}$: p ≤ 0.01, $^{\blacklozenge\blacklozenge\blacklozenge}$: p ≤ 0.001 (compared to avHPAI H5N8/636099); ^{x}: p ≤ 0.05, ^{xxxx}: p ≤ 0.0001 (compared to avHPAI H5N8/642613); $^{\Delta\Delta\Delta\Delta}$: p ≤ 0.0001 (compared to avLPAI H5N8/MP5883).
